# Prediction of contraceptive discontinuation among reproductive-age women in Ethiopia using Ethiopian Demographic and Health Survey 2016 Dataset: A Machine Learning Approach

**DOI:** 10.1186/s12911-023-02102-w

**Published:** 2023-01-17

**Authors:** Shimels Derso Kebede, Yakub Sebastian, Abraham Yeneneh, Ashenafi Fentahun Chanie, Mequannent Sharew Melaku, Agmasie Damtew Walle

**Affiliations:** 1 Department of Health Informatics, School of Public Health, College of Medicine and Health Science, Wollo University, Dessie, Ethiopia; 2 Department of Information Technology, College of Engineering, IT and Environment, Charles Darwin University, Darwin, Australia; 3 Department of Health Informatics, Institute of Public Health, College of Medicine and Health Science, University of Gondar, Gondar, Ethiopia; 4 Department of Health Informatics, Institute of Public Health, College of Medicine and Health Sciences, Mettu University, Mettu, Ethiopia

**Keywords:** Contraceptive discontinuation, Machine learning, Association rule mining, Ethiopian Demographic and Health Survey, Ethiopia

## Abstract

**Background:**

Globally, 38% of contraceptive users discontinue the use of a method within the first twelve months. In Ethiopia, about 35% of contraceptive users also discontinue within twelve months. Discontinuation reduces contraceptive coverage, family planning program effectiveness and contributes to undesired fertility. Hence understanding potential predictors of contraceptive discontinuation is crucial to reducing its undesired outcomes. Predicting the risk of discontinuing contraceptives is also used as an early-warning system to notify family planning programs. Thus, this study could enable to predict and determine the predictors for contraceptive discontinuation in Ethiopia.

**Methodology:**

Secondary data analysis was done on the 2016 Ethiopian Demographic and Health Survey. Eight machine learning algorithms were employed on a total sample of 5885 women and evaluated using performance metrics to predict and identify important predictors of discontinuation through python software. Feature importance method was used to select top predictors of contraceptive discontinuation. Finally, association rule mining was applied to discover the relationship between contraceptive discontinuation and its top predictors by using R statistical software.

**Result:**

Random forest was the best predictive model with 68% accuracy which identified the top predictors of contraceptive discontinuation. Association rule mining identified women's age, women’s education level, family size, husband’s desire for children, husband’s education level, and women’s fertility preference as predictors most frequently associated with contraceptive discontinuation.

**Conclusion:**

Results have shown that machine learning algorithms can accurately predict the discontinuation status of contraceptives, making them potentially valuable as decision-support tools for the relevant stakeholders. Through association rule mining analysis of a large dataset, our findings also revealed previously unknown patterns and relationships between contraceptive discontinuation and numerous predictors.

## Background

Contraceptive method discontinuation reduces the contraceptive utilization coverage and family planning program effectiveness, as well as contributes to undesired fertility [1]. Contraceptive discontinuation can be reduced by increasing the variety of methods available, as well as providing clients with more information, technical competence, interpersonal relationships, follow-up and continuity mechanisms, and the appropriate constellation of services, all of which contribute to overall care quality [2]. To better understand and take action against this problem, machine learning algorithms could be used to predict and discover significant predictors of contraceptive discontinuation. Machine learning is one of the most rapidly evolving fields of artificial intelligence used in many areas of life, primarily in the healthcare field [3].

Even though family planning programs have increased contraceptive use, globally, 38% of contraceptive users discontinue the use of a method within the first 12 months [4]. Evidence from the World Health Organization (WHO) study on 60 countries' demographic and health survey (DHS) showed that the percentage of accidental pregnancies due to contraceptive discontinuation lies in the range of 5% to 20% [5]. The discontinuation of contraceptives has been reported as significantly more common in sub-Saharan Africa (SSA) [4, 6] where the discontinuation rate was higher than 50% in most countries [7]. In Ethiopia, more than one-third of all contraceptive users (35%) discontinued use within 12 months and three months after discontinuation, 42% or more women were at risk of conception [5, 8]. Findings from various studies in Ethiopia also reported that contraceptive discontinuation for all methods was higher ranging from 23% for Implanon to 69% for long-acting reversible contraceptives [9–17].

A high proportion of contraceptive discontinuation is a public health problem frequently associated with unintended pregnancies, unwanted births, and unsafe abortions, which have increased risks of pregnancy and childbirth-related maternal morbidity [18, 19]. In countries with moderate-high contraceptive prevalence, most unintended pregnancies occur when contraceptive methods are discontinued or used inappropriately [9, 20]. Consequently, unintended pregnancy negatively affects a woman's psychological well-being by increasing perinatal depression, stress, and lower levels of life satisfaction [21–24].

The Ethiopian Federal Ministry of Health (FMOH) aims to increase the contraceptive prevalence rate to 50% by 2025 [25]. Ethiopia also planned to ensure availability of quality and safe family planning information and services to decrease unmet need for family planning from 22 to 17% by 2030. However, evidence indicated that 35% of women with an unmet need for modern contraception have used a modern method of contraception in the past but have chosen to discontinue. This shows high rates of discontinuation poses a threat to the achievement of these targets. Several variables could influence the choice to proceed or abandon the continual use of contraceptives including age group, education, area of residence, household economic status, family size, parity, television and radio ownership, and decision-maker to use contraceptives [10, 14, 26]. A greater understanding of these variables will inform policymakers, programmers, and other partners to minimize discontinuation rates and reinforce family planning interventions to attain the Sustainable Development Goals (SDGs) targeted at reducing maternal and under-5 child mortality by 2030. Predicting the risk of discontinuing contraceptives is also used as an early-warning system to notify family planning programs. Moreover, Machine learning (ML) offers a potential mechanism for identifying important factors associated with various health outcomes for public health researches.

Various research studies have been conducted to study the potential factors associated with contraceptive discontinuation by applying classical statistical analysis techniques [20, 27–30]. However, no previous studies have tried to predict contraceptive discontinuation and identify predictor factors with the application of machine learning. Due to this reason, previously used statistical techniques become less precise as the number of input variables and possible associations increases [31] which leads to unreliable results. To overcome these problems, machine learning algorithms are appropriate as they can improve the accuracy of prediction over conventional regression models by capturing the complex and nonlinear relationships in the data [32]. Hence, this study aimed to predict contraceptive discontinuation and identify its predictors using the current state-of-the-art machine learning models.

## Methodology

The study was conducted using secondary data from Ethiopian Demographic and Health Survey (EDHS) 2016 dataset. EDHS is a population-based cross-sectional study design that was carried out from January 18, 2016, to June 27, 2016, in Ethiopia. Ethiopia have been conducted nationally representative household surveys every five years to collect information for a variety of population overall health, nutritional, maternal, and child health issues [8]. Hence, the 2016 EDHS collected a nationwide data that allowed the calculation of key demographic indicators such as contraceptive discontinuation.

### Source and study population

#### Source population

All women aged 15–49 in Ethiopia whoever used contraceptives within the five years prior to the EDHS 2016 survey.

#### Study population

All women aged 15–49 in the selected enumeration areas whoever used contraceptives within the 5- years prior to EDHS 2016 survey.

### Sample size determination

In the Ethiopian DHS 2016, a total of 18,008 households were selected for the sample, and a total of 16,650 occupied households were successfully interviewed, yielding a response rate of 98%. 16,583 eligible women were identified for interviews from the households, but it was conducted with 15,683 women, achieving a 95% response rate [8]. The sample in this analysis is weighted to adjust for non-response and variations in probability of selection. Furthermore, the sample used is limited to responses from women who had ever used any contraceptive method within the five years before the survey. Thus, the analysis was restricted to a weighted sample of 6,737 reproductive-age women. The data for predicting contraceptive discontinuation is obtained from a woman’s questionnaire.

### Study variables

#### Dependent variable

Contraceptive discontinuation status was the dependent variable which dichotomized into two categories as discontinued and not-discontinued.

#### Independent variable

The independent variables were adopted from previous studies [12, 14, 27, 33–35] and includes age of women, residence, marital status, religion, women occupation, sex of household head, region, employment, women education, husband education, internet usage, mobile ownership, wealth index, owning TV, owning radio, khat chewing, alcohol drinking, smoking cigarette, fertility preference, parity, family size, husband’s desire for a child and woman’s abortion history.

#### Operational Definition

Contraceptive discontinuation was computed from the reproductive calendar which consists of two columns; column 1) Births, pregnancies, terminations and contraceptive use and column 2) Reasons for discontinuation of contraceptive use. For the end of each episode of contraceptive use recorded in column 1 of the calendar, the interviewer asks additional questions to ascertain the reason for discontinuing use of any contraceptive method (modern/traditional) and records the code for the reason for discontinuation in column 2 of the calendar in the row corresponding to the month of ending use of the method. Discontinuation of a method is determined if the box for the month in the first column specified any method use and column 2 specified any reason to discontinue a contraceptive. However, if column 2 is blank/not filled any value it shows continuation.

During variable recoding, this information was obtained from V302A (Ever used anything or tried to delay or avoid getting pregnant) and V360 (Reason of last discontinuation). So, if a woman ever use any contraceptive method and she mentioned a reason for last discontinuation of method, then she assumed to be a discontinuer. But if she didn't mention anything for a reason for last discontinuation of method, she assumed to be a non-discontinuer.

### Data processing and analysis

This study employed the general framework used in a prior study [36] built based on Yufeng Guo's 7 Steps of Machine Learning [37] to predict contraceptive discontinuation. In his article, Yufeng Guo outlines the seven steps in supervised machine learning namely: Data collection, Data preparation, Model selection, Model training, Model evaluation, parameter tuning, and making a prediction. Machine learning algorithms for this study were implemented using scikit-learn [38] version 1.1.1, and xgboost [39]version 1.6 packages in python using Jupyter Notebook.

#### Data collection

The dataset for this study is available on the Measure Demographic and Health Survey website and obtained upon a formal request. The data contains a weighted sample of 6,737 reproductive-age women who had ever used a method of both modern and traditional contraception.

#### Data preparation/pre-processing

The process of transforming data, which includes data cleaning, exploratory data analysis, normalization, and dimensionality reduction, can have a profound impact on the model's performance. Data preparation techniques that were employed in this study were data cleaning, feature engineering, dimensionality reduction, and data splitting.

##### Data cleaning

Data Cleaning is the first step after the data is retrieved and consists of detecting and removing outliers, handling missing values, and handling unbalanced categories of the outcome variable from the dataset. In this study, k-nearest neighbors (KNN) imputation technique was used to impute missing values of independent variables in the dataset. In a previous study [40], KNN imputation provided a more robust and sensitive method for missing value estimation. Another data cleaning task was imbalanced data handling. Imbalanced data is a dataset in which the values of the outcome variable is dominated by one category, while the other category is underrepresented. Machine learning models trained on imbalanced data are typically biased toward the majority class and fail to predict cases that are rare/minority class [41]. The problem of imbalanced data is currently well recognized and there are various approaches to address data imbalance [42].Hence, a random oversampling technique such as Synthetic Minority Oversampling Technique (SMOTE) [43] was used to balance the training data.

##### Feature engineering

The process of transforming raw data into features that better represent the underlying problem to predictive models, resulting in improved model accuracy on unseen data, is known as feature engineering [44]. Among various feature engineering techniques, encoding of categorical variables into numeric values known as one hot encoding for nominal variables and label encoding for coding each category of variables as a number was done through preprocessing module of the scikit-learn package.

##### Dimensionality reduction

Dimensionality reduction refers to reducing the number of input variables for a predictive model. Fewer input variables can lead to a simpler predictive model, which can perform better when making predictions on new data [45]. There are two approaches to dimension reduction: feature selection and feature extraction, with the latter being more appropriate for pattern recognition or image processing [36]. Feature selection involves using statistics to assess the relationship between the independent input variables and the outcome variable and selecting independent variables that have the highest importance to predict the target variable. Hence, feature selection was made through the feature importance technique (which is implemented in the feature importance property of ML models) to identify the most important predictors of contraceptive discontinuation. Feature importance is the average measure of how significant a feature is in comparison to other features used in the ensemble model to predict the outcome variable; higher feature importance means that that feature is used to differentiate one outcome versus the other more frequently [46]. This technique has been widely used in previous public health studies to identify predictors/factors of various health outcomes [46–49]. Furthermore, it has been demonstrated to be the best feature selection method when compared to other feature selection methods such as Boruta and recursive feature elimination techniques, which makes it extremely useful and efficient in selecting the important variables [50].

##### Data split

Every machine learning algorithm needs training and test/validation data to train the model and validate it on data it has never seen before. A simple 80/20 split method in which 80% of the data is used for training and the remaining 20% for testing the model was used. However, tenfold cross-validation method was used in this study for model training as it does not waste a lot of data, which is a big advantage when the number of samples is small [38]. K-Fold divides all observations into equal-sized groups of samples called folds and k-1 folds used to train the prediction function, and then the fold that is left out is used for testing k times repeatedly [38]. The k-fold cross-validation performance measure is the average of the values computed in the loop.

#### Model selection

After the data has been prepared and divided into training and testing tests, suitable models were selected to perform the training. Since the outcome variable was categorical, the task was a classification task and appropriate classifiers need to be selected to conduct the prediction. The dataset used in the analysis fall under the category of binary classification since contraceptive discontinuation was categorized into two mutually exclusive categories. The classification algorithms used for this analysis were logistic regression (LR), Random forest (RF), KNN, artificial neural network, support vector machine (SVM), Naïve Bayes, eXtreme gradient boosting (XGBoost), and AdaBoost classifiers. These algorithms were selected based on previous studies which applied machine learning techniques for classification tasks on EDHS data [46–48, 51–53].

#### Model training

Following model selection, the selected classifiers were trained with balanced and unbalanced data and their performance was compared through tenfold cross-validation. After comparison, the best predictive model was selected and trained with balanced training data for the final prediction on unseen test data.

#### Model evaluation

Model evaluation was performed after the model has been trained to determine how well it is performing by testing the model's performance on previously unseen data that was reserved for this purpose during data splitting. The confusion matrix, which is a simple cross-tabulation of the actual and predicted categories for the outcome variable, is a common method for analyzing the performance of a classification model [54]. Confusion metrics can be used to calculate performance metrics such as overall accuracy, precision, recall, and F1 score, which will all be used in this study to compare the performance of the selected classifiers. Furthermore, the ROC curve (or receiver operating characteristic curve) was used for visualizing the performance of ML models.

The confusion matrix and different derived metrics are adapted from [55] and presented as shown in Table 1.

**Table 1:** Confusion matrix

Actual	Predicted	
	Positive prediction	Negative prediction
Positive Class	True positive (TP)	False negative (FN)
Negative Class	False positive (FP)	True negative (TN)

Accuracy = TP + TN / (TP + FP + FN + TN)

Precision = TP / (TP + FP)

Recall = TP / (TP + FN)

F1 score = (2 * Precision * Recall) / (Precision + Recall)

#### Hyperparameter tuning

A model Hyperparameter is an external configuration to the model whose value must be specified by the user as it can't be estimated from data [56]. A simple example of a hyperparameter is the number of neighbors (K) in the K-nearest neighbor algorithm which should be specified manually. For this study, hyper-parameter tuning was done through Optuna [57] framework. According to the authors, Optuna works by formulating the hyperparameter optimization as a process of minimizing/maximizing an objective function that takes a set of hyperparameters as an input and uses the Bayesian framework to understand better the probability of the optimal values and avoid unnecessary computation for the combination of non-performing parameters in the search for the optimal parameter settings. This framework is better than traditional hyperparameter tuning techniques such as grid search and randomized search which takes explicitly defined hyperparameters by the user and uses only those hyperparameters to optimize the model.

#### Making prediction

This is the final stage of the machine learning approach in which all the above activities come into action. Prediction is estimating the outcome variable based on independent variables. In this case, contraceptive discontinuation was determined based on important variables identified along the way. Given different predictor variables, whether a woman will discontinue or not her contraceptive use was determined based on the best performing classifier with a specified accuracy.

Even though machine learning analysis identified the most important predictors, it doesn’t show which category is more associated with the presence of discontinuation. In this study, association rule analysis was applied through the Apriori algorithm (arules package) through R software (version 4.2.1) to identify a specific category of predictor variables that have associations with contraceptive discontinuation. Association rules are IF–THEN rules which are very important as they are easy to interpret and they select only the relevant features for the model during rule generation [58]. Instead of using straightforward tests of statistical significance, association rule mining (ARM) can find strong and frequent relationships between variables based on measures of "interestingness" which are related to the effect size of a pattern [59].

An association rule is a pair (X, Y) of sets of attributes, denoted by x → Y, where x is the left-hand side of the rule /antecedent and Y is the right-hand side/consequent of the rule which states that if X happens, then Y would also happen. It is a fundamental data mining technique that thoroughly searches for frequent patterns, correlations, and associations among the sets of variables making them ideal for discovering predictive rules from medical data repositories [60, 61]. It has been used in prior healthcare research to identify risk factors for various health outcomes such as early childhood caries [62], parasite infection [63], motorcycle crash casualty [64], stroke [65], and to discover symptom patterns of coronavirus disease of 2019 (COVID-19) [66].

**Fig. 1:**
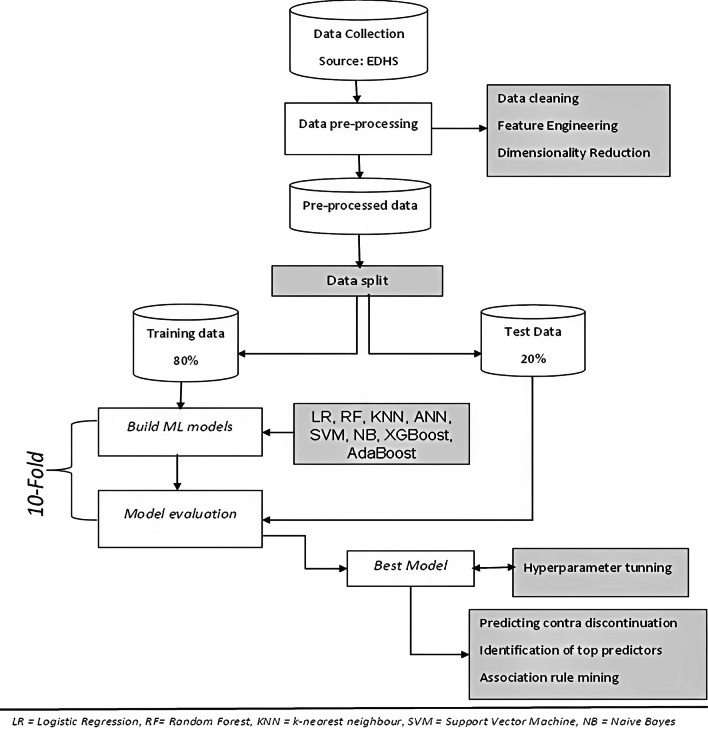
Overview flow chart of data preparation and analysis plan applied

The strength of an association rule can be measured by support (the prevalence of both X and Y co-occurring), confidence (the probability that Y occurs given that X is already present), and lift [67] which refers to the deviation of the support parameter from what would be expected if X and Y were independent. Confidence also called the accuracy of association rules is an indication of how often the rule has been found to be true /how reliable the rule is [68]. If the lift is less than one, the appearances of X and Y are negatively correlated, which implies that if one is present, the other is likely to be absent, and if it is greater than one, x and Y are positively correlated, which indicates that if one is present, the other is likely to be present as well. However, X and Y are independent and have no relationship if the lift value of the rule equals one [65]. Finally, the overall data preparation and analysis workflow is presented in Fig. 1.

## Results

### Characteristics of participants

#### Sociodemographic characteristics

A total weighted sample of 6737 reproductive-age women was included in the study with a mean age of 30.3 years (± 7.79 standard deviation) and the majority of women, 2558 (37.97%) were in the age group between 26 and 34 years. The majority of participants were rural residents 5101 (75.79%) and married or living with their partners 5925 (87.95%). Over half, 3622 (53.76%) of the participants were Orthodox Christians followed by 1571 (23.32%) Muslims. About 5562 (82.56%) women lived in male-headed households with about half of the participants 2917 (43.29%) being a housewife. Regarding the regional distribution of respondents, about one-third of 2221 (32.96%) were from the Amhara region followed by 2037 (30.24%) from the Oromia region and 1304 (19.35%) from the Southern Nations, Nationalities, and Peoples’ Region (Table 2).

**Table 2:** Sociodemographic characteristics of reproductive age women in Ethiopia, EDHS 2016

Variable	Categories	Weighted Frequency	Percentage
Women’s Age	15 to 25	2114	31.38
	26 to 34	2558	37.97
	35 to 49	2065	30.65
Residence	Urban	1631	24.21
	Rural	5106	75.79
Religion	Orthodox	3621	53.76
	Protestant	1461	21.68
	Muslim	1571	23.32
	Others	83	1.24
Marital Status	Single	192	2.84
	Married or living with partner	5925	87.95
	Others	620	9.21
Household head sex	Male	5562	82.56
	Female	1175	17.44
Women Occupation	Not working/Housewife	2917	43.29
	Professional	304	4.51
	Agricultural/Farmer	1661	24.66
	sales or services	1330	19.74
	Manual or other	525	7.79

#### Socio-economic characteristics

More than half 3568 (52.96%) of the respondents were without formal education and only 2081 (30.89%) had primary education. The majority of respondents also lived in a household without radio ownership 4523 (67.14%), TV ownership 5514 (81.86%), and two-thirds of 4211 (62.51%) respondents were unemployed. About half of the respondents lived in rich households, however, 4811 (71.41%) had no mobile phone and almost all 6440 (95.59%) of the participants were non-internet users (Table 3).

**Table 3:** Socioeconomic characteristics of reproductive age women in Ethiopia, EDHS 2016

Variable	Categories	Weighted Frequency	Percentage
Educational level	No formal education	3568	52.96
	Primary education	2081	30.89
	Secondary or Higher	1088	16.15
Radio ownership	No	4523	67.14
	Yes	2214	32.86
TV ownership	No	5514	81.86
	Yes	1223	18.14
Wealth status	Poor	1944	28.86
	Middle	1395	20.71
	Rich	3397	50.43
Uses Internet	No	6440	95.59
	Yes	297	4.41
Employed	No	4211	62.51
	Yes	2526	37.49
Owns mobile	No	4811	71.41
	Yes	1926	28.59

#### Fertility related characteristics

More than half 3940 (58.49%) of the respondents lived in households of size with less than 5 members and about one-third 2311 (34.30%) of participants had a parity of 1 to 2. The majority of respondents also wanted to have another child 3785 (56.18%) and 6056 (89.91%) never aborted during the five years prior to EDHS 2016 (Table 4).

**Table 4:** Fertility-related characteristics of reproductive age women in Ethiopia, EDHS 2016

Variable	Categories	Weighted Frequency	Percentage
Parity	None	806	11.96
	1 to 2	2311	34.30
	3 to 4	1796	26.67
	5 and more	1823	27.07
Fertility preference	Want to have other	3785	56.18
	Wants no more	2551	37.87
	Undecided	401	5.95
Family size	5 or less	3940	58.49
	Above 5	2797	41.51
History of abortion	No	6057	89.91
	Yes	680	10.09

#### Behavioral characteristics

Among the total respondents, about 3078 (45.70%) were alcohol drinkers. However, a small number of 837 (12.44%) respondents were khat chewers and almost all 6681 (99.18%) women were non-smokers (Table 5).

**Table 5:** Behavioral characteristics of reproductive age women in Ethiopia, EDHS 2016

Variable	Categories	Weighted Frequency	Percentage
Chewing chat	No	5899	87.56
	Yes	838	12.44
Alcohol drinking	No	3658	54.30
	Yes	3079	45.70
Smoking cigarette	No	6681	99.18
	Yes	56	0.82

#### Regional distribution and types of discontinued methods

**Fig. 2:**
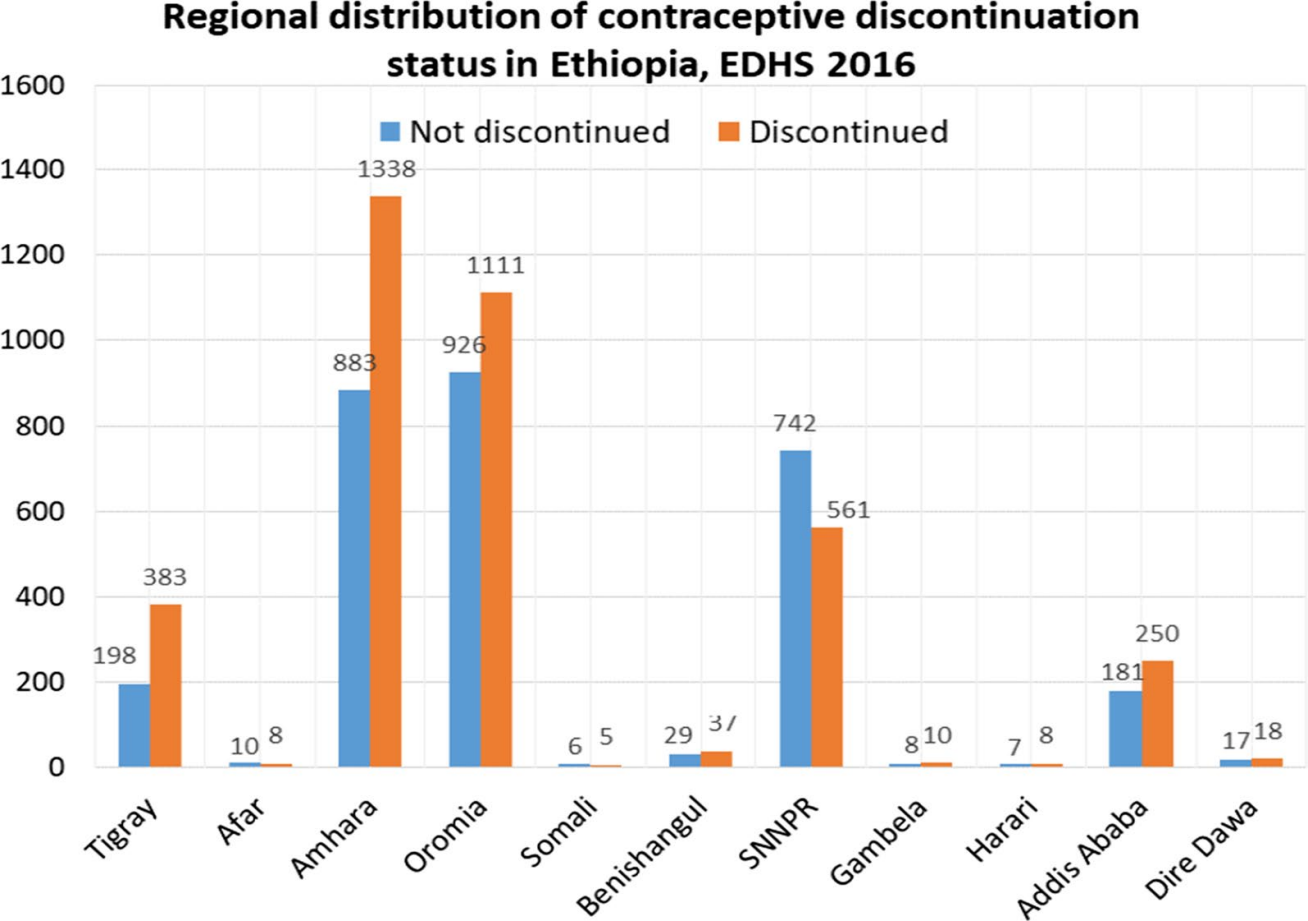
Regional distribution of contraceptive discontinuation status in Ethiopia, EDHS 2016

**Fig. 3:**
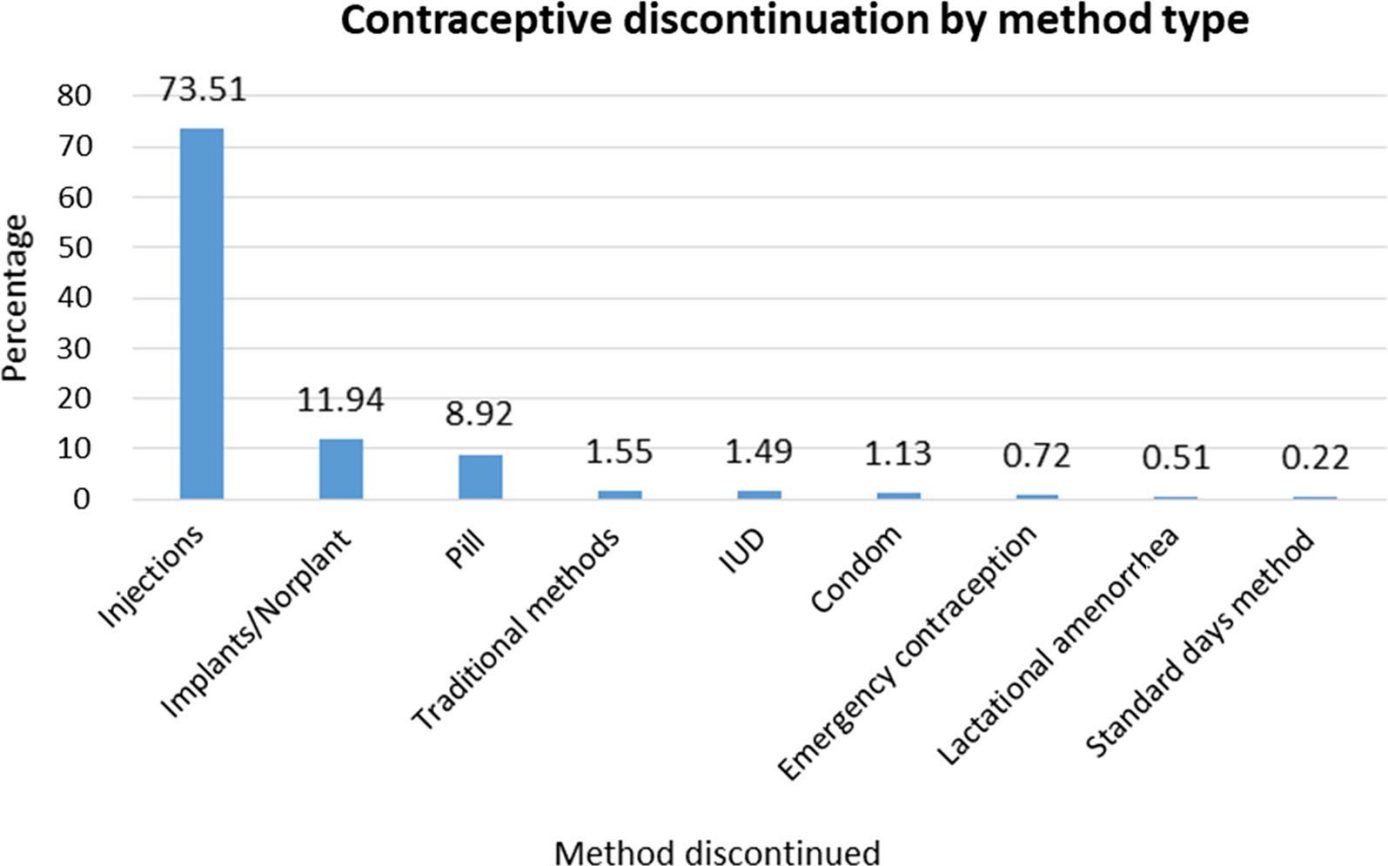
Contraceptive discontinuation by method type, EDHS 2016

The number of discontinuation cases was highest in the Amhara region, Oromia region, Tigray region, and Addis Ababa city administration (Fig. 2). The highest contraceptive discontinuation was reported among injection users (73.5%), and the lowest was observed among standard days method users (0.22%) (Fig. 3).

### Machine learning analysis of contraceptive discontinuation

#### Balancing data

**Fig. 4:**
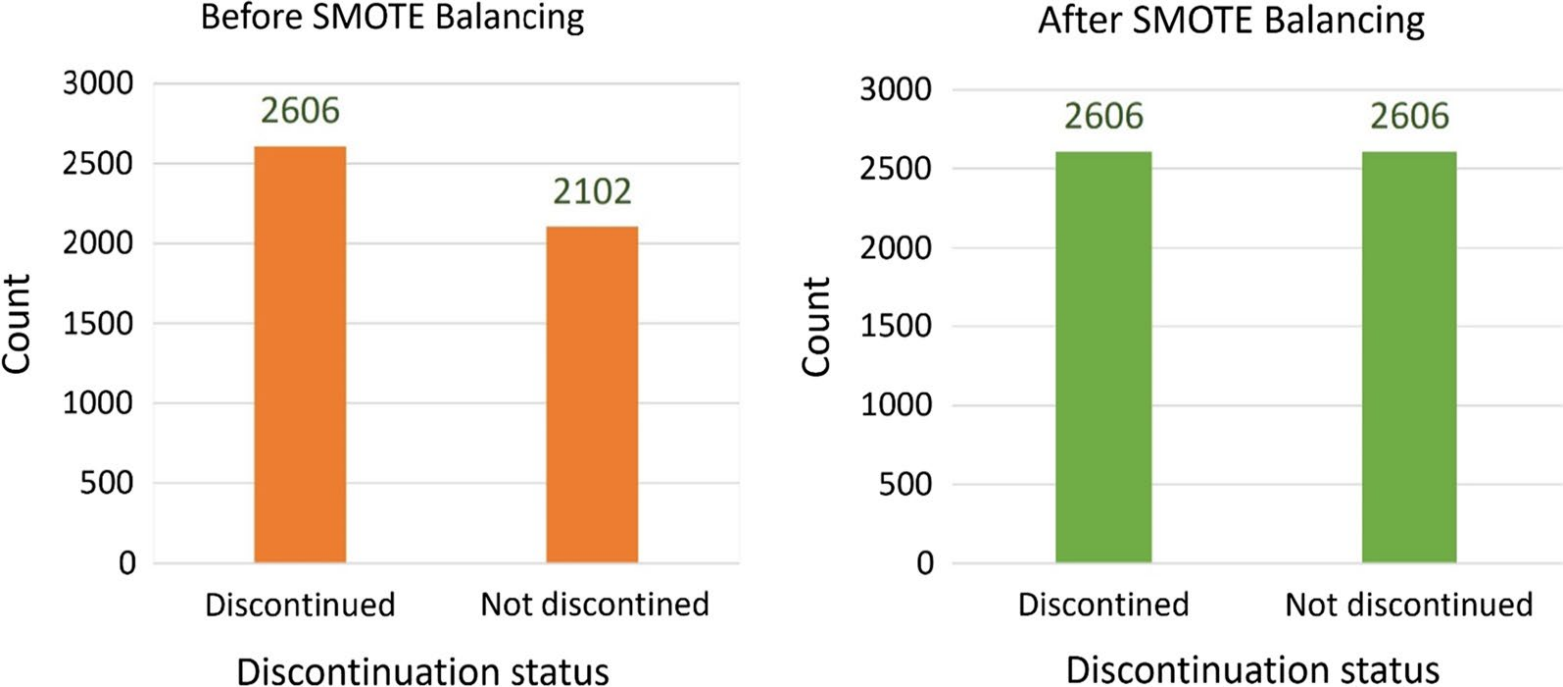
Distribution of contraceptive discontinuation status before and after balancing

Learning from class-imbalanced data is common and challenging problem in supervised learning as standard classification algorithms are designed to handle balanced class distributions. Hence, to balance the skewed distribution of the outcome variable SMOTE oversampling generated 504 additional synthetic observations from minority class. As a result, the overall distribution of contraceptive discontinuation was changed from original skewed to symmetric distribution for both classes (Fig. 4).

#### Feature selection

Variable importance feature selection technique is a method for determining the most important predictors of an outcome variable, similar to how p-values and t-statistics are used in most traditional statistical approaches, such as linear regression and logistic regression, to determine which variables are significant [69]. Feature importance is the average measure of how important a feature is in comparison to other features used in the ensemble model to predict the outcome [46]. Higher feature importance indicates that the feature was used to distinguish one outcome from another more frequently and is a relative measure.

**Fig. 5:**
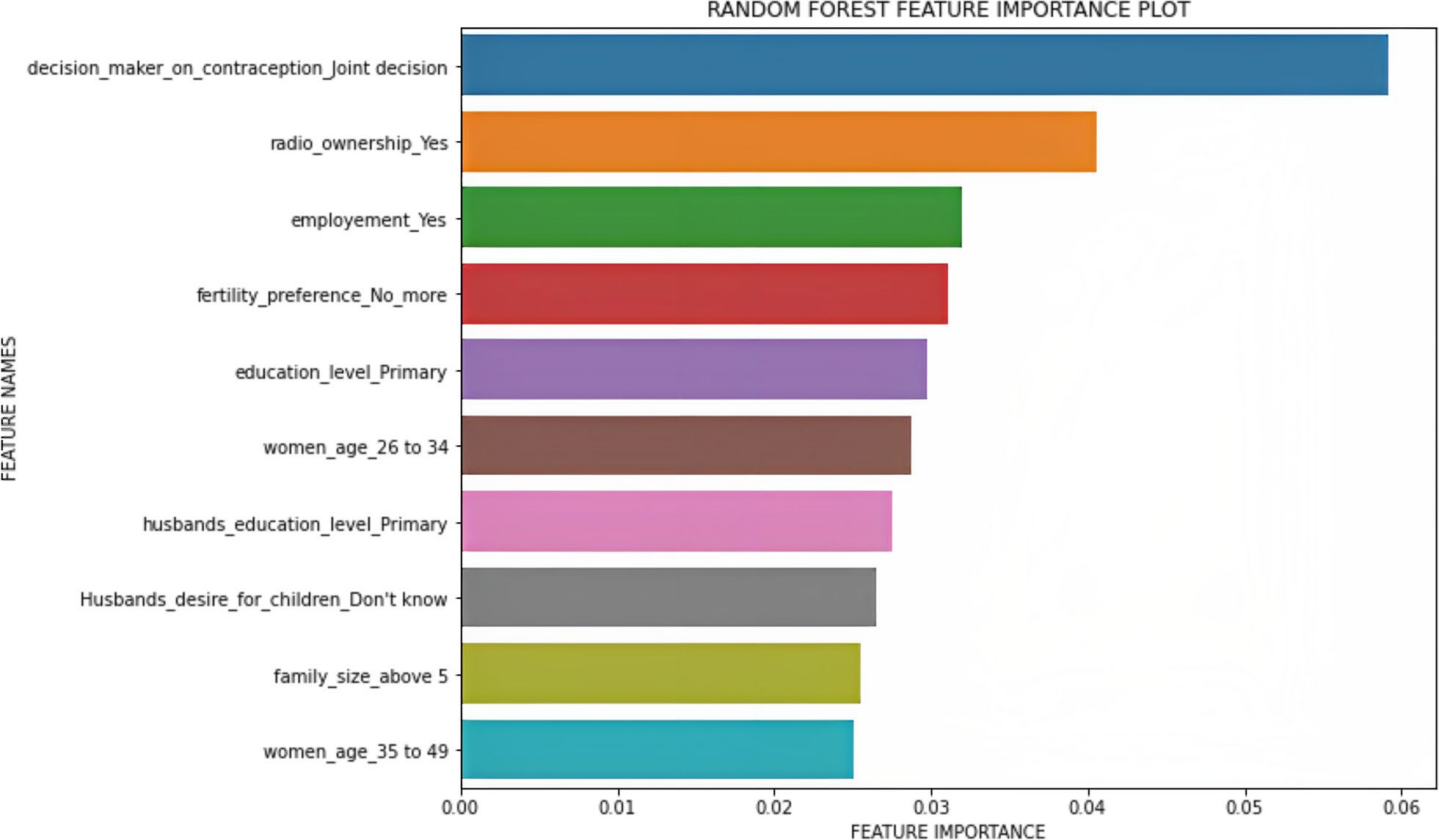
Feature importance plot for predictors for contraceptive discontinuation in Ethiopia, EDHS 2016

According to random forest feature importance results, decision-makers on contraceptive use, radio ownership, women’s employment status, and fertility preference were the top five most important predictors of contraceptive discontinuation. Furthermore, women’s age, husband’s education level, husband’s desire for children, and family size were also important predictors of contraceptive discontinuation (Fig. 5). The x axis (length of the bars) shows the relative importance of independent variables in predicting contraceptive discontinuation. The longer the bar, the feature is more important in predicting whether a woman discontinue contraceptive use or not.

#### Predictive modelling

After model selection and training, the mean accuracy and mean area under the curve (AUC) score of ML models in stratified tenfold cross-validation were used to compare the performance of predictive models to predict contraceptive discontinuation. After comparing classifiers through stratified tenfold cross-validation on the unbalanced training data, the support vector machine was the best model with an accuracy of 65% and 68.4% area under the receiver operating characteristic (ROC) curve. However, this result is misleading due to the unbalanced nature of the outcome variable. After balancing the training data using SMOTE oversampling technique, the model comparison was done and random forest was the best predictive model with an accuracy of 67.9% and 74.1% area under the ROC curve (Table 6).

**Table 6:** Model comparison through cross-validation on training data

ML Model	Data	Accuracy score (%)	AUC score
Dummy classifier	Unbalanced	49.9	0.49
	Balanced	50.9	0.50
Random Forest	Unbalanced	63.8	0.65
	Balanced	67.9*	0.74*
Logistic Regression	Unbalanced	63.5	0.67
	Balanced	65.6	0.71
KNN	Unbalanced	59.8	0.60
	Balanced	61.9	0.66
SVM	Unbalanced	64.9	0.68
	Balanced	58.5	0.70
AdaBoost	Unbalanced	63.5	0.67
	Balanced	66.2	0.72
XGBoost	Unbalanced	62.2	0.65
	Balanced	67.1	0.72
Artificial Neural Net (MLPClassifier)	Unbalanced	60.7	0.63
	Balanced	65.1	0.71
Naïve Bayes (GaussianNB)	Unbalanced	59.4	0.61
	Balanced	59.1	0.63

*Maximum Performance

#### Hyperparameter tuning of random forest

Among multiple hyperparameters of random forest algorithm number of trees in the forest(n_estimators), number of features for the best split(max_features), minimum number of samples required to split an internal node(min_samples_split), minimum number of samples required to be at a leaf node(min_samples_leaf), and number of samples to draw from independent variables to train each tree(max_samples) were optimized on a given search space to maximize the mean accuracy using stratified 10- fold cross validation with one hundred trials. The default parameter and optimized hyperparameters are shown in Table 7.

**Table 7:** Default and optimal tuned hyperparameters of Random Forest Model

Hyperparameter	Default	Optimal value
n_estimators	100	200
max_features	Square root of the number of features	0.15
min_samples_split	2	2
min_samples_leaf	1	2
max_samples	None	0.96

#### Predicting contraceptive discontinuation

**Fig. 6:**
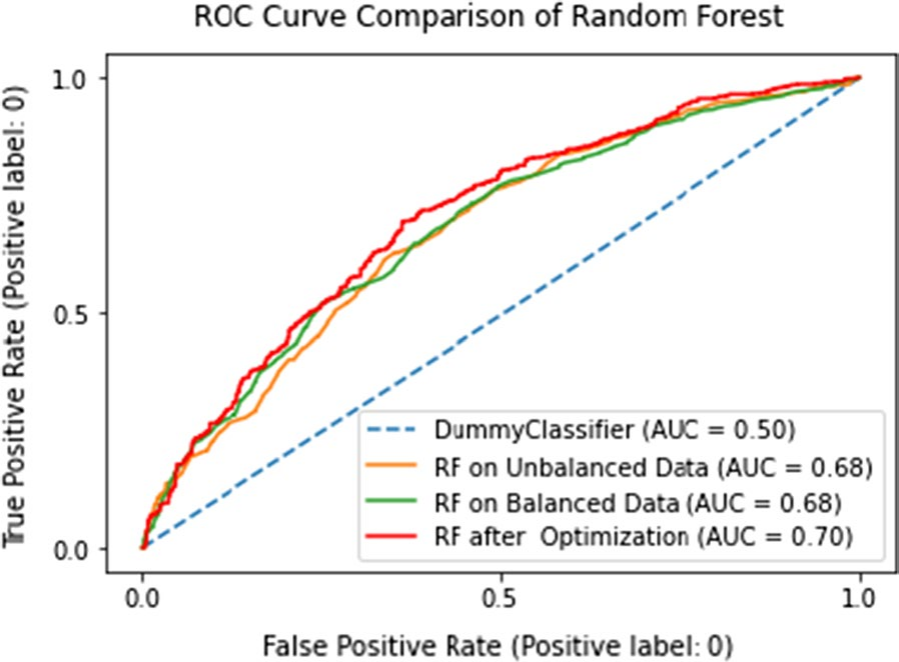
Comparison of random forest model prediction on test data

After selecting the best model, prediction of contraception discontinuation was done on previously unseen test data. The prediction was made after training random forest on unbalanced training data, balanced data with default model parameters, and compared with an optimized model trained with balanced data. After training the random forest model on unbalanced and balanced data, the prediction on unseen test data provided area under ROC curve score of 0.68 for both. However, hyperparameter-tuned random forest predicted a better AUC of 0.7. The comparison is shown in Fig. 6.

**Fig. 7:**
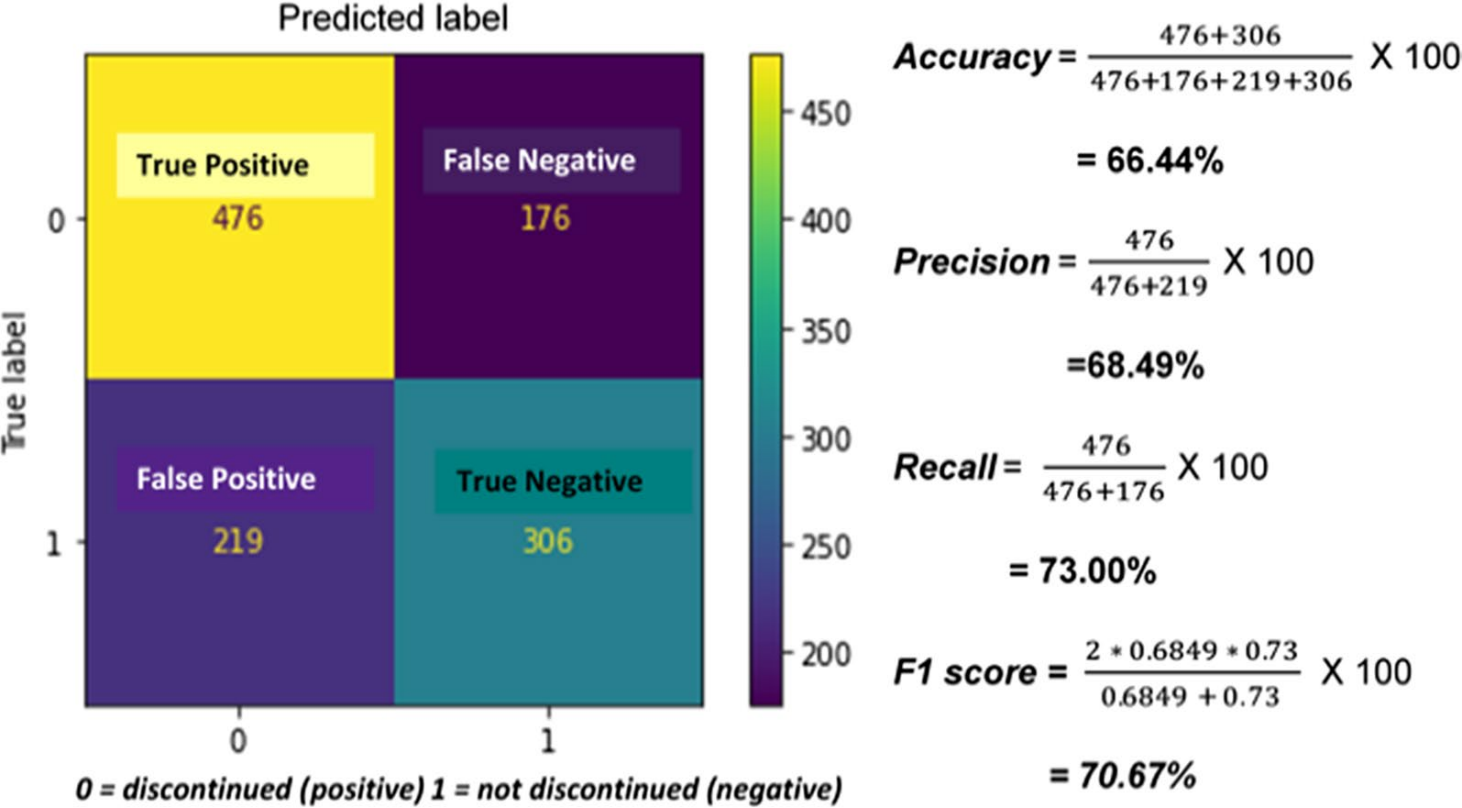
Confusion matrix of Random Forest prediction on test data

The performance of the hyperparameter tuned random forest model was tested on 1177 test samples after training. Out of 652 true discontinued cases, the model predicted 476 of them correctly as discontinuers (true positive). And out of 525 non discontinuers, the model predicted 306 of the as true non discontinuers (true negative). But the model, misclassified 219 non discontinuers as discontinuer (false positive) and 176 true discontinuers as non-discontinuer. Overall, the model predicted with an accuracy of 66.4%, precision of 68.5%, F1 score of 70.6%, and 73% recall on test data (Fig. 7).

### Association rule mining

The Apriori algorithm generates 11 rules. Among those antecedents (factors) the most common factors strongly associated with contraceptive discontinuation were women's age (25 to 34), women’s education level (primary), family size (5 and below), and husband's desire for children (Husband wants fewer children).

Furthermore, husband education (primary) and fertility preference (women prefer to have another child) were also most frequently associated with a high probability of contraceptive discontinuation.

**Fig. 8:**
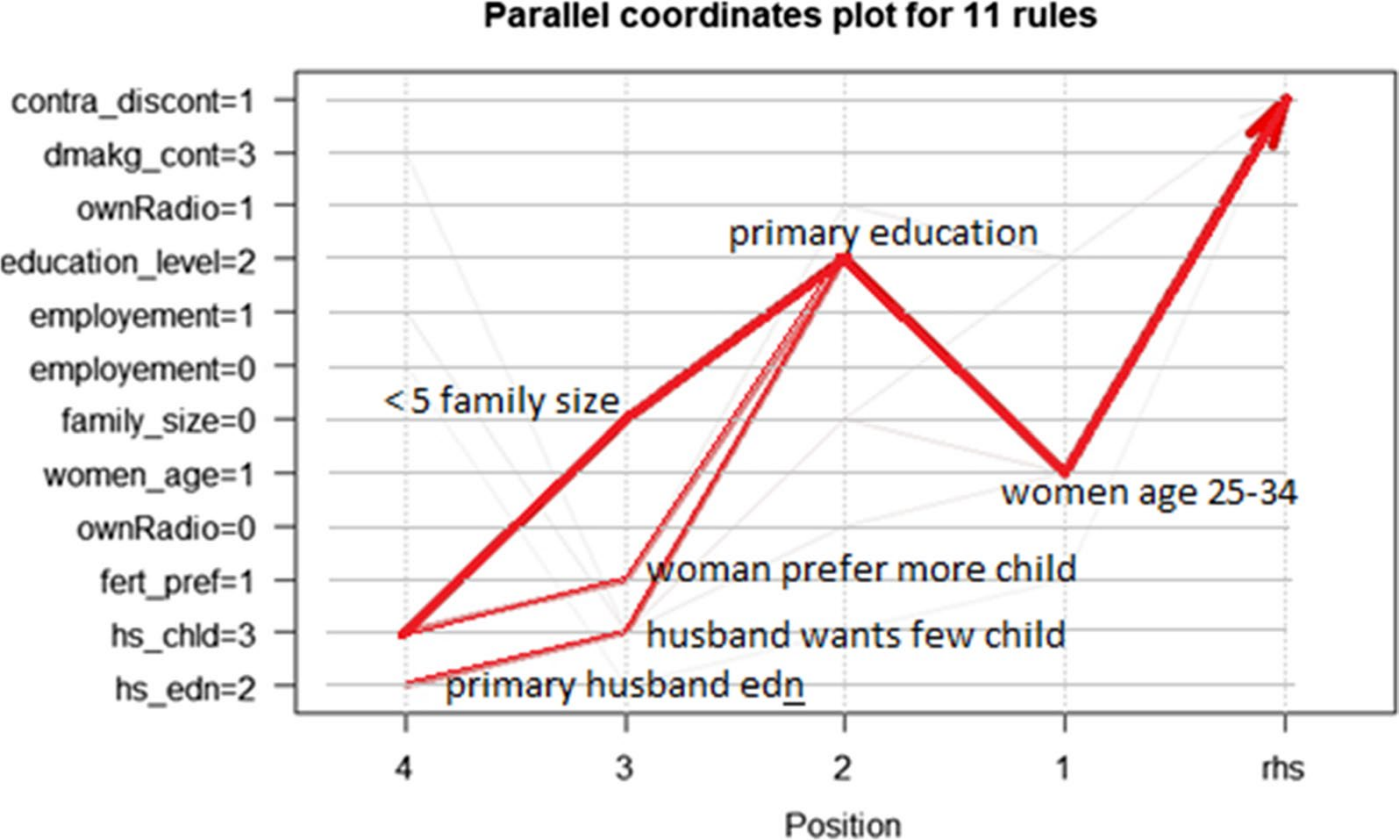
Parallel coordinates plot of association rules

Figure 8 shows the parallel coordinate plot for all eleven rules. The thick and bold red color of the lines means the variable is highly associated with a higher probability of contraceptive discontinuation, whereas fuzzy red and invisible lines mean the variable is unlikely to increase the probability of discontinuation. The x-axis shows the position of variables in the rule.

The top five association rules based on their lift values are listed with their correspondent lift value and the probability of contraceptive discontinuation are listed below.

- **Rule 1. If** women age = 1(aged 25 to 34), Education level = 2(primary education)**,** Family size = 0 (less than 5), her husband’s desire for children = 3 (husband wants few), **Then** probability of discontinuation is 94.4% (lift = 1.7).
- **Rule 2. If** women age = 1, education level = 2, her husband’s desire for children = 3, her husband’s education level = 2(primary education), **Then** the probability of discontinuation is 88.9% (lift 1.6).
- **Rule 3:** If women age = 1, education level = 2, fertility preference = 1 (prefer to have another child), her husband’s desire for children = 3, **Then** probability of discontinuation is 88.6% (lift = 1.6).
- **Rule 4.** If women age = 1, family size = 0, her husband’s desire for children = 3, husbands’ education level = 2, **Then** probability of discontinuation is 81.8% (Lift = 1.5).
- **Rule 5. If** education level = 2, Radio ownership = 1(household has radio), Fertility preference = 1, Husband’s desire for children = 3, Husbands’ education level = 2, **Then** probability of discontinuation is 81.1% (Lift = 1.465).

## Discussion

This study was conducted to predict and identify the top predictors of contraceptive discontinuation in Ethiopia. For this purpose, eight machine learning algorithms were trained on both balanced and imbalanced data via tenfold cross-validation. The performance of those eight ML models was compared by their classification accuracy and ROC score. Consequently, SMOTE balancing technique provided a higher accuracy and ROC score than models trained on imbalanced data. Furthermore, the Random Forest model was the best predictive model with an accuracy of 68% and ROC 0.74 based on a tenfold cross-validation score on balanced training data. This finding was comparable to a study conducted in Ethiopia, which found Random Forest as the best machine learning model to predict nutritional status for under-five children using EDHS data, with accuracy and AUC of 68.2% and 0.76 respectively[48]. However, the performance was much lower than a study conducted in Indonesia [51] which reported AdaBoost as the best model to predict the duration of contraceptive use with an accuracy of 85.1%. This inconsistency could be due to the size of the dataset used for model building. This study used only 5885 records whereas the Indonesian study used 39,594 records, which allows the model to learn effectively and perform the prediction with better accuracy.

The final prediction on the test data was made after optimizing hyperparameters of the random forest algorithm which in turn improved the accuracy of the model. The optimized model predicted 476 discontinued cases correctly, and 176 non-discontinued cases incorrectly from a total of 652 discontinued cases on the unseen test data. The random forest model was the best predictive model for this study with 66.4% accuracy, 68.5% precision, 73% recall, and 70.8% f1 score performance on the unseen test data.

As another aim of this research to identify predictors of contraceptive discontinuation, feature importance techniques of random forest identified the top ten predictors with the highest relative importance. Hence, decision-makers on contraceptive use, radio ownership, women’s employment status and, fertility preference, women’s age, husband’s education level, husband’s desire for children, and family size were the most important factors which predict contraceptive discontinuation in Ethiopia.

Association rule mining was applied on important predictors of contraceptive discontinuation identified by the best model, to discover important patterns and associations of those factors with the outcome variable. The result of ARM revealed that middle women age (25 to 34), education level of the women being primary, below five family size, husband desire for children (Husband wants fewer children), husband’s education being primary, and fertility preference of the women (prefer to have another child) were most frequently associated with a high probability of contraceptive discontinuation.

ARM results demonstrated that women in their early reproductive years (25–34 years) more often discontinued the use of contraceptives. This result was supported by studies conducted in Bangladeshi [26], six countries DHS (Bangladesh, Philippines, Kazakhstan, Dominican Republic, Kenya, and Zimbabwe) which showed that women who had discontinued contraception were somewhat concentrated in the 25–34 ages [18], and a study in Ethiopia which stated that woman whose age is between 25 and 34 had a higher risk of discontinuing modern contraceptive methods [70]. This may be due to the reason thinking of reaching the menopausal period in the older mothers [9] which may urge them to have a child as early as possible.

Women with primary education levels were more likely to experience discontinuation. This result was similar to studies conducted in Ethiopia [12, 71], three west African countries [1], and Turkey [72] which stated that women having their higher educational level were more likely to discontinue their contraceptives than those women who had no formal education. This would be because educated women may seek health information and have better access to information regarding their contraceptive methods which in turn makes them look for more effective methods. As per a study conducted in Texas United States, women who wanted to use a more effective method had a higher risk of discontinuation [73]. This claim was also supported by a study conducted in Dilla Town, Southern Ethiopia, which reported a higher risk of method switching among women who have attended primary education as compared to women who do not attain formal education [74]. However, this finding is inconsistent with studies done in Diguna Fango district of Welayta zone, southern Ethiopia, and public health facilities in central Ethiopia which reported that women not having formal education were more likely to discontinue their Implanon use [34, 75]. The possible explanation for this inconsistency could be the difference in contraceptive methods studied. In this study, all types of methods were included whereas only the Implanon method was studied in studies conducted in Diguna Fango district, public health facilities in central Ethiopia.

This study also revealed that women with low family sizes were more likely to discontinue their contraceptive use. This result was in line with studies conducted in Ethiopia [9, 75] which stated high discontinuation of contraceptives in small-sized households. Since the economic status of women is very much linked to the discontinuation of contraceptives [76], this result may be attributed to a desire of women to increase their household size by having more children as they become economically strong through time.

Another factor for contraceptive discontinuation was the fertility preference of a woman. Those women who prefer to have another child often discontinue their contraceptives. This result was in line with finding from a study conducted in Butajira town, Ethiopia which reported that five times higher likelihood of discontinuation for women who desire to have more children [77]. This may be related to the fact that majority of the respondents in this were middle-aged women. Women at this age would be resourceful and could have what it takes to raise a child, which causes them to prefer more children by discontinuing contraceptive use.

The husband’s education level was also important factors associated with contraceptive discontinuation. Women whose husbands had primary education had a higher likelihood of discontinuation. This result was supported by a study conducted in Bangladesh [78]. This may be because educated husbands may have better access to information on possible side-effects of current and alternative choices of contraceptive methods used by their wives, which in turn leads to switching to other suitable methods by discontinuing their current methods.

Women whose husbands desired to have fewer children were more likely to discontinue their contraceptive use. This may be due to women’s autonomy in which they decide about their use of contraceptives by themselves which means their husbands wouldn’t force them to discontinue as long as the woman wanted to continue. This factor was also identified as a statistically significant factor for discontinuation in a previous study conducted in three West African countries which reported that a husband’s desire for more children was associated with a higher likelihood of discontinuation in contraceptive methods [1]. This inconsistency could be due to a difference in the method of analysis since this study used the association rule mining technique which is good at discovering unusual relationships between variables, whereas the other study used traditional logistic regression analysis.

According to a previous study [14] done on similar study participants women’s education, residence, women having no children, husband desire for children, women’s self-decision when using a contraceptive, decision making for using a contraceptive, discussion about family planning with a healthcare worker, and information about side effects were factors significantly associated with contraceptives discontinuation. Even though some predictors were similar to the previous study, some factors such as women’s age, family size, husband’s education, and women’s fertility preference were revealed by this study as having previously unseen relationships with contraceptive discontinuation. This is one of the benefits of machine learning and data mining analysis techniques to discover unknown relationships in a dataset. However, there is a discrepancy between the two studies regarding other factors being associated with the outcome variable. The disagreement may be due to the nature of the analysis methods applied. Classical statistical methods are based on a series of assumptions which usually hard to fulfill. While machine learning analysis doesn’t make any assumptions on data distribution which makes it preferable to explore the actual relationship between the variables within the dataset. Hence, the previous study has applied multilevel logistic regression, a classical statistical analysis which a have a series of assumptions to be fulfilled.

## Limitation and strength of the study

Our findings should be considered in light of some limitations. First, the parameters are difficult to interpret and quantify the strength of their association with the dependent variable since ML algorithms lack regression coefficients for each predictors. Second, dependent variable was computed using a DHS contraceptive calendar, which is a month-by-month retrospective history of all births, pregnancies, abortions, and contraceptive use episodes of each surveyed woman experienced in the five years prior to being interviewed. As a result, the participants may have difficulty in memorizing their past experiences, and it may be challenging to calculate contraceptive discontinuation. Last not but least, we were unable to investigate additional variables related to contraceptive discontinuation because our study relied on secondary data.

Nevertheless, this study has some strengths. Despite the fact that machine learning results have interpretation limitations due to their black-box nature, this study used further analysis to reveal the effect of identified predictors, whether they increase or decrease discontinuation of contraceptive use. It also revealed previously unidentified hidden patterns and relationships in the area. Furthermore, this study provides an invaluable contribution to contraceptive use and discontinuation literature in the context of machine learning.

## Conclusions

This study was aimed to predict and identify predictors of contraceptive discontinuation. Hence eight ML algorithms were trained and evaluated their performance based on accuracy and area under the curve to predict contraceptive discontinuation. Random forest RF was the best predictive model with an accuracy of 68% and ROC 0.74 based on tenfold cross-validation. Top ten important predictors of contraceptive discontinuation were identified through the random forest feature importance method and those predictors were used in association rule mining to explore their patterns and relationships with contraceptive discontinuation. Finally, association rules identified middle women age (25 to 34), education level of the women being primary, below five family size, husband desire for children (Husband wants fewer children), husband’s education being primary, and fertility preference of the women (prefer to have another child) as most frequently associated factors with higher probability of contraceptive discontinuation. Our findings demonstrated that the machine learning classification algorithms under consideration can accurately predict the discontinuation status of contraceptives, making them potentially valuable as decision-support tools for the relevant stakeholders. Through association rule mining analysis of a large dataset, our findings also revealed previously unknown patterns and relationships between contraceptive discontinuation and numerous predictors. Furthermore, women’s age, education level, family size, and their husband characteristics must all be taken into consideration while implementing health policies intended to decrease the discontinuation of contraceptives in Ethiopia.

## Data Availability

All relevant data are in the manuscript. However, the minimal data underlying all the findings in the manuscript will be available upon request. EDHS (2016) data was used which is available on the public domain through the Measure DHS website (www.measuredhs.com).

## Abbreviations

ARM: Association rule mining
AUC: Area under the curve
COVID-19: Coronavirus disease of 2019
DHS: Demographic and Health Survey
EDHS: Ethiopian Demographic and Health Survey
FMOH: Ethiopian Federal Ministry of Health
FN: False negative
FP: False positive
KNN: K-nearest neighbor
LR: Logistic regression
ML: Machine learning
RF: Random forest
ROC: Receiver operating characteristic
SDG: Sustainable Development Goals
SMOTE: Synthetic Minority Oversampling Technique
SSA: Sub-Saharan Africa
TN: True negative
TP: True positive
SVM: Support Vector Machine
WHO: World Health Organization
XGBoost: EXtreme gradient boosting
